# Foliar application of various biostimulants produces contrasting response on yield, essential oil and chemical properties of organically grown sage (*Salvia officinalis* L.)

**DOI:** 10.3389/fpls.2024.1397489

**Published:** 2024-06-28

**Authors:** Davide Farruggia, Giuseppe Di Miceli, Mario Licata, Claudio Leto, Francesco Salamone, Johannes Novak

**Affiliations:** ^1^ Department of Agricultural, Food and Forest Sciences, Università degli Studi di Palermo, Palermo, Italy; ^2^ Research Consortium for the Development of Innovative Agro-Environmental Systems (CoRiSSIA), Palermo, Italy; ^3^ Clinical Department for Farm Animals and Food System Science, University of Veterinary Medicine, Vienna, Austria

**Keywords:** antioxidant, essential oil, medicinal and aromatic plant, organic, phenolic, yield

## Abstract

Sage (*Salvia officinalis* L.) is a medicinal and aromatic plant (MAP) belonging to the *Lamiaceae* family. Its morphological, productive and chemical characteristics are affected by abiotic and biotic factors. The use of biostimulants seems to be one of the most interesting innovative practices due to fact they can represent a promising approach for achieving sustainable and organic agriculture. Despite a large application in horticulture, the use of biostimulants on MAPs has been poorly investigated. On this basis, a field experiment in a 2-year study was done to assess the effect of foliar treatments with different types of biostimulants (containing seaweeds, fulvic acids and protein hydrolysates) and two frequencies of application on morphological, productive, and chemical characteristics of *S. officinalis* grown organically in Mediterranean environment. Morphological, productive, and chemical parameters were affected by the factors. The biostimulant application generated higher plant height, chlorophyll content, relative water content, biomass yield and essential oil yield compared to control plants. In addition, more frequent application of biostimulants produced higher biomass and essential oil yield. The application of fulvic acid and protein hydrolysates every week produced the highest total fresh yields (between 3.9 and 8.7 t ha^-1^) and total dry yields (between 1.3 and 2.5 t ha^-1^). The essential oil yield almost doubled (33.9 kg ha^-1^) with a higher frequency of protein hydrolysates application. In this study, 44 essential oil compounds were identified, and the frequency factor significantly influenced the percentage of 38 compounds. The highest percentage of some of the most representative monoterpenes, such as 1,8-cineole, α-thujone and camphor, were observed in biostimulated plants, with average increases between 6% and 35% compared to control plants. The highest values for total phenolics, rosmarinic acid, antioxidant activity were obtained in control plants and with a lower frequency of biostimulant applications. This study emphasizes how biostimulant applications may be used to improve sage production performance and essential oil parameters when produced in agricultural organic system. At the same time, biostimulants application caused a decrease in total phenolic, antioxidant activity and rosmarinic acid values.

## Highlights

- Sage yield is significantly affected by the type of biostimulant.
- Fulvic acids and protein hydrolysates produce the best performance in terms of biomass yield.
- Protein hydrolysates are found to increase the essential oil yield the most.
- Total phenolics, antioxidant activity and rosmarinic acid are negatively affected by biostimulants.
- Biostimulants permit to obtain appreciated yields in organic agriculture.

## 1 Introduction

Medicinal and aromatic plants (MAPs) are a source of bioactive chemicals and are known for their distinct flavor and aroma (Sharifi-Rad et al., 2017; Bernardini et al., 2018). They were also among the first treatments employed by humans to alleviate illnesses (Giannenas et al., 2020; Ovidi et al., 2021). The *Lamiaceae* family is one of the largest families of MAPs and *Salvia* is the largest genus in the *Lamiaceae* family, with about 900 species spread throughout Europe, Asia, Africa, and America (Nazar et al., 2022; Schmiderer et al., 2023).

Producer interest in *Salvia* species has increased recently due to its commercial importance. The leaves of this species are utilized as a raw material in the food, pharmaceutical, medicinal, and perfume industries (Rahmani Samani et al., 2019; Tuttolomondo et al., 2020). Due to their potent antibacterial, antifungal, antioxidant, anticancer, anticholinesterase, and anti-inflammatory qualities as well as their ability to boost mood and cognitive function, *Salvia* species have also attracted the attention of scientists studying their biological characteristics (Fu et al., 2013; Rahmani Samani et al., 2019; Aslani et al., 2023). *Salvia* species are particularly rich in steroids, polyphenols, triterpenoids, diterpenoids, and sesquiterpenoids (Ovidi et al., 2021; Mot et al., 2022).

The essential oil (EO) of *S. officinalis* is known for its remarkable variability in the main monoterpene constituents β-pinene, 1,8-cineole, α-thujone, β-thujone and camphor (Soltanbeigi et al., 2021; Schmiderer et al., 2023).

Numerous elements, including genetic traits, climatic circumstances, environment organisms, applied agro-techniques, and post-production processing, influence the quality and production of secondary metabolites in MAPs (Figueiredo et al., 2008; Méndez-Tovar et al., 2016; Kulak et al., 2020; Soltanbeigi et al., 2021; Tuttolomondo et al., 2021; Farruggia et al., 2024). The cultivation of medicinal plants using suitable and recommended agricultural practices, such as those concerning fertilization and irrigation, can achieve optimal agronomic output and supply the industry with standard bioactive compounds (Sotiropoulou and Karamanos, 2010; Rahmani Samani et al., 2019; Khammar et al., 2021; Farruggia et al., 2023). In addition, according to several authors (Yadav et al., 2014; Morshedloo et al., 2017; Mohammadi-Cheraghabadi et al., 2021), prolonged water stress conditions during MAP developmental stage may cause changes in physiological and metabolic processes as well as having a negative impact on transpiration and photosynthetic processes, which results in a significant decline in growth and yield performance.

MAPs can coexist with organic farming methods, which are popular among both growers and buyers (Lubbe and Verpoorte, 2011; Kosakowska et al., 2019; Najar et al., 2021; Farruggia et al., 2024). Biological fertilizers are applied as an alternative to chemical fertilizers in organic and sustainable agricultural systems to boost soil fertility, soil organic matter and plant development (De Pascale et al., 2017; Ye et al., 2020). Using bio-based fertilizers in agriculture is a viable and effective way to increase production stability and nutrient usage efficiency, even in less-than-ideal circumstances (Chojnacka et al., 2020; Puglia et al., 2021). Many biostimulants are bio-based products (Calvo et al., 2014; Corsi et al., 2022);. Biostimulants can influence both primary and secondary plant metabolism and improve the plants’ ability to tolerate adverse soil pH, heat, salinity, drought, and nutritional stress (Ertani et al., 2013; Colla et al., 2014; Lucini et al., 2015; Di Miceli et al., 2023; Farruggia et al., 2024).

Due to the fact that biostimulants include macro- and micronutrients, sterols, polysaccharides, betaines, and additional enhancing-promoting compounds, a number of studies (Briceño-Domínguez et al., 2014; Hernandez-Herrera et al., 2014; Arioli et al., 2015; Rouphael et al., 2017; Consentino et al., 2023; Sabatino et al., 2023) have shown that their application can improve agronomic, productive, and qualitative response as well as the absorption of water and nutrients and the photosynthetic process. Furthermore, these compounds can improve the amount of photosynthetic pigments, carotenoids, total phenols, and NPK concentration (Colla et al., 2014; Malécange et al., 2023; Sabatino et al., 2023). They also have a good effect on several molecular processes, such as protein synthesis, photosynthetic activity, and enzyme activity (Zanin et al., 2019; Nardi et al., 2021).

Previous studies (Rouphael et al., 2017; Kocira et al., 2020; Del Buono, 2021) showed that biostimulants have been linked to lower production costs and higher product quality; using them could be a financially viable way for farmers to satisfy consumer expectations and rising industry demands for sustainability and environmental protection. At the same time, biostimulants may contribute to a decrease in the number of agricultural inputs used, which would lower production costs (Bulgari et al., 2015; Del Buono, 2021; Shahrajabian et al., 2021). Studies conducted in many nations (Rouphael et al., 2017; Popko et al., 2018; Elansary et al., 2019; Rahimi et al., 2022; Di Miceli et al., 2023; Franzoni et al., 2023); demonstrate the positive effects of biostimulants on yield and the quality traits of open-field and greenhouse crops. However, studies concerning the effect of the foliar application of biostimulants on the productive and qualitative parameters, and on the EO profile of cultivated sage are lacking. Information on the topic is limited to one type of foliar biostimulant and one frequency of application, which were the focus in the very few research studies that have been carried out to date (Rahmani Samani et al., 2019; Skrypnik et al., 2022).Based on this, the goal of this study was to assess how four distinct biostimulants and two frequencies of application affected morphological, productive, and chemical characteristics of *S. officinalis* L. grown organically in a Mediterranean climate without irrigation. In particular, the following hypothesis were tested: 1) the foliar application of biostimulants improves productive parameters in sage plants cultivated in open field; 2) the foliar application of biostimulants affects the chemical parameters of the extract depending on the type of biostimulant and frequency of application.

## 2 Materials and methods

### 2.1 Experimental site and plant material

Tests were carried out at a local farm located in Aragona (Sicily, Italy) (330 m a.s.l., 37°22′32.71″ N, 13°38′33.59″ E), during the growing seasons of 2022 and 2023. The soil was classified as Regosol (United States Department of Agriculture (USDA) classification: typic xerorthents) and sandy clay loam (46% sand, 27% clay and 27% silt) with a pH of 7.3, 14 g kg^−1^ organic matter, 1.25% total nitrogen, 19.8 ppm assimilable phosphate, and 358 ppm assimilable potassium. Agamic propagation was carried out. The seedlings were produced by a commercial nursery and grown in plastic pots for 60 days. The plants were transplanted at the beginning of spring 2021. The distance between rows and within rows was 2.00 m and 0.50 m, respectively. Sage plants were managed under rainfed conditions. Before transplanting, the experimental field received organic fertilization through the distribution of 2 t ha^−1^ of manure which was buried at a depth of 0.30 m. No pesticides or chemical fertilizer were used in either year. Weeds were removed mechanically at the end of winter and before harvesting.

### 2.2 Weather data

**Figure 1:**
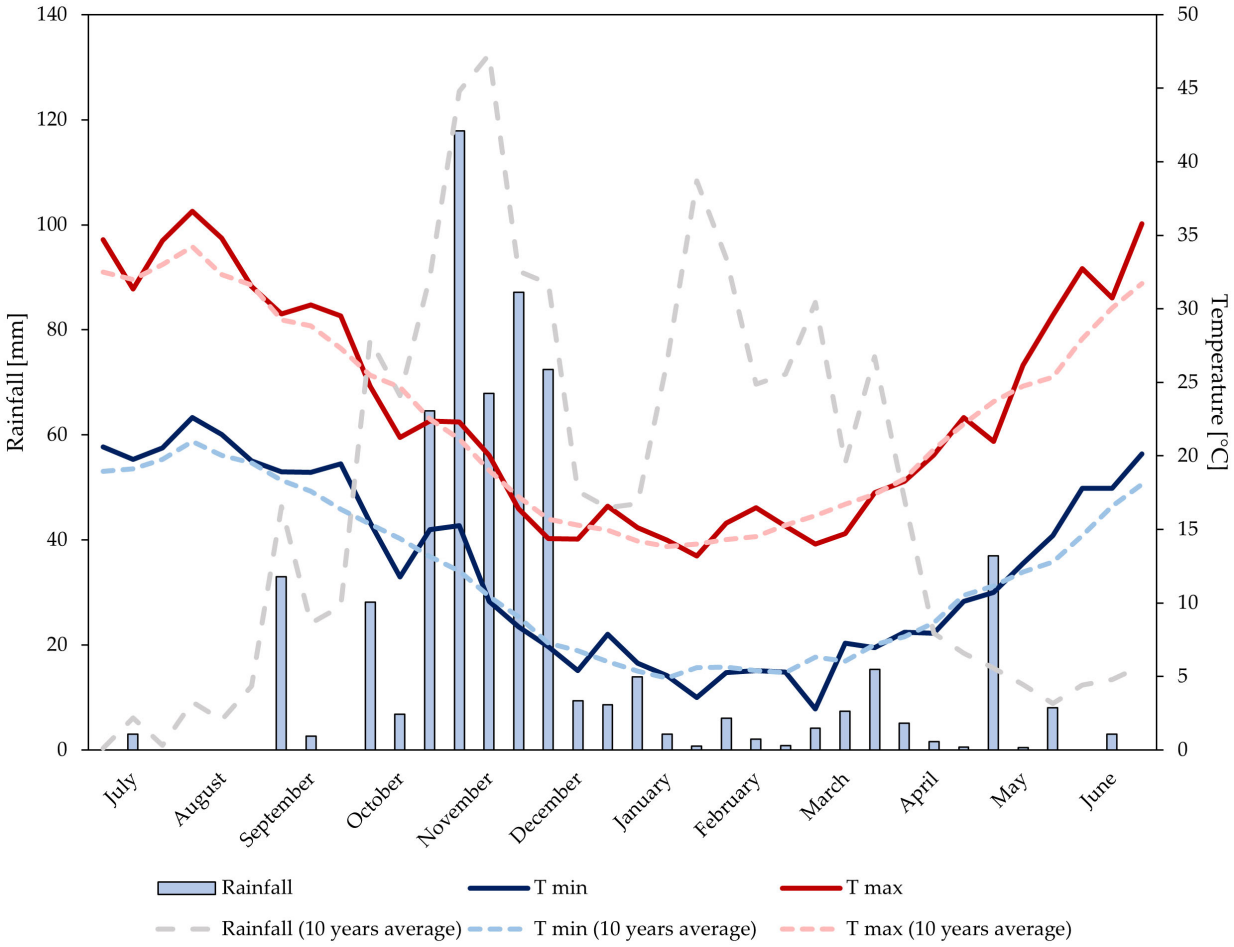
Temperature and rainfalls trends at the experimental site during growing season 2021–2022.

**Figure 2:**
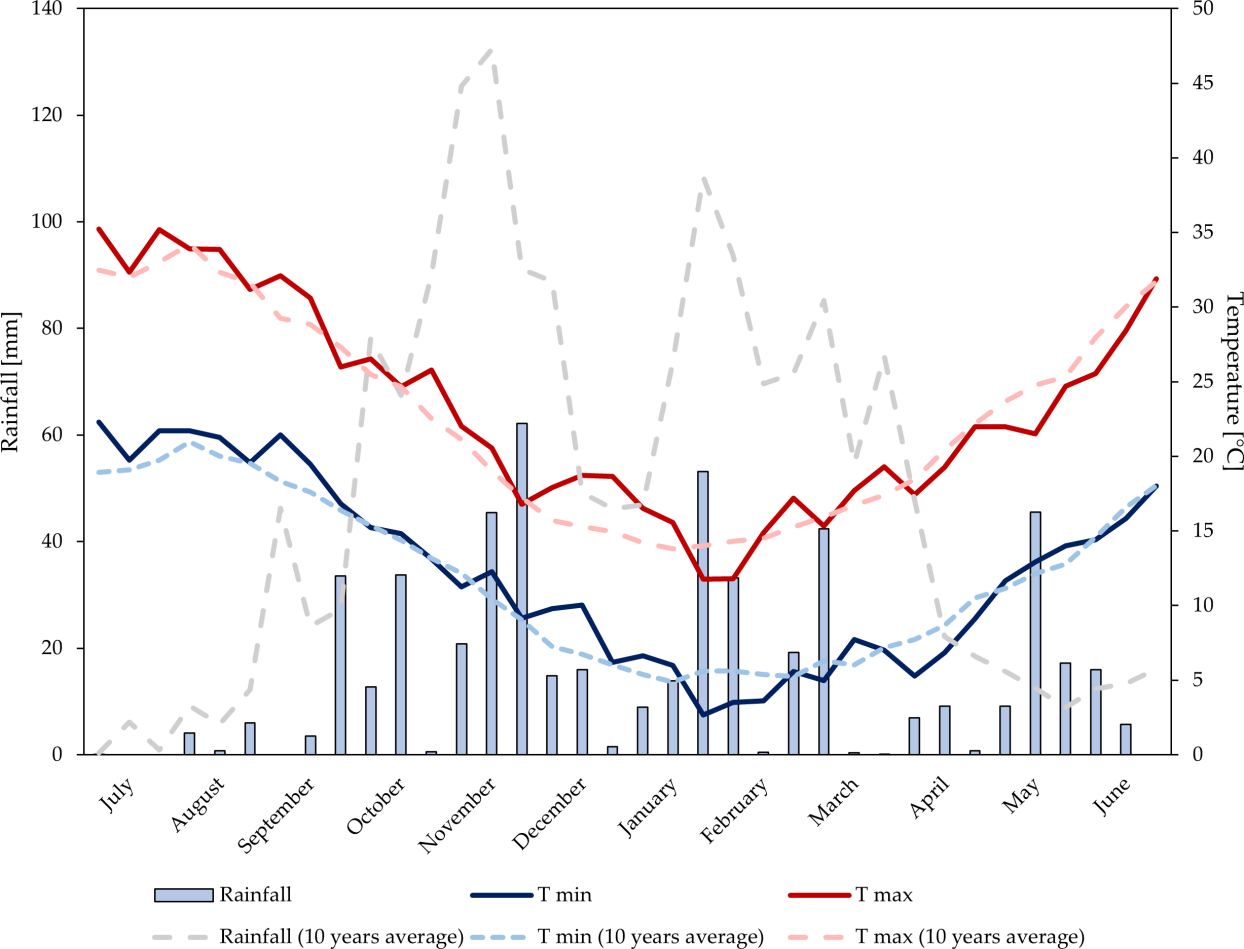
Temperature and rainfalls trends at the experimental site during growing season 2022–2023.

Data on rainfall and air temperature were recorded at a weather station belonging to the Sicilian Agro-Meteorological Information Service (SIAS, 2023). The station is equipped with a datalogger and various sensors for the measurement of air temperature (TAM platinum PT100 sensor, heat resistance with anti-radiation screen) and total rainfall (PPR sensor with tilting bucket rain gauge). Data regarding average daily maximum and minimum temperatures (°C) and total 10-day period precipitation (mm) were taken into consideration. Air temperature and rainfall trends are shown in **Figures 1**, **2**.

Over the two years, annual rainfall levels were 611 mm (2021–2022), and 538 mm (2022–2023). In the first growing season, rainfall was mainly distributed from October to December. During the period of biostimulant application and until the harvest, a total rainfall of 56 mm was observed. Significant rainfall occurred in the first 10-day period of May (37 mm). In the second growing season, rainfall was well-distributed from the end of September to March. During the period of biostimulant application and until the harvest, double rainfall levels (110 mm) compared to the first year were observed. Significant rainfall occurred in the second 10-day period of May (45 mm). In both years, temperatures trends were similar and consistent with the average temperature of the experimental area.

### 2.3 Treatments

Four commercial biostimulant formulations provided by the company “Mugavero fertilizers” were used for the tests.

- Protein Hydrolysate (PH), obtained from *Fabaceae* and containing amino acids and plant peptides (31%), organic nitrogen (5%) and organic carbon (25%).
- *Ecklonia maxima* (EM), containing organic nitrogen (1%), organic carbon (10%), auxin (11 ml l^−1^) and cytokinin (0.03 mg l^−1^), and organic substances with nominal molecular weights< 50 kDa (30%).
- *Ascophyllum nodosum* (AN), containing organic nitrogen (1%), organic carbon (10%), phytohormones and organic substances with nominal molecular weights< 50 kDa (30%).
- Fulvic Acids (FA), extracted from leonardite and containing organic nitrogen (0.5%) and organic carbon (30%).

The biostimulant dosage was planned to provide the same total amount of N, considering the N content of each type of product. The same total amount of biostimulant were applied following two frequencies of application, weekly frequency (F1) for a total of six applications and two-weeks frequency (F2) with three total applications. The doses are listed in **Table 1**.

**Table 1:** Doses of foliar biostimulants.

Biostimulant	Dose^1^ Frequency 1 week [l hl^-1^]	Dose^1^ Frequency 2 weeks [l hl^-1^]	Total Amount^2^ [l ha^-1^]
PH = Protein hydrolysate	0.025	0.050	1.2
EM = *Ecklonia maxima*	0.125	0.250	6.0
AN = *Ascophyllum nodosum*	0.125	0.250	6.0
FA = Fulvic acids	0.250	0.500	12.0

^1^ = doses for each application; ^2^ = total quantity of biostimulant applied.

The first application was performed during the first week of April in each year. For each foliar application, 4 hl of water ha^−1^ were used. A portable sprayer with an operating pressure of 250 kPa was adopted. A randomized complete block design with three replicates was used for the tests. Biostimulant (B) and frequency (F) were used as fixed effects in the linear model/ANOVA. Each block comprised 10 plots of 30 m^2^. Treatments were applied for each randomized plot in the block. The plots were well spaced in the block; plastic panels were used to delimit each plot and to avoid drift during foliar applications.

### 2.4 Plant measurement

At harvest, plant height, chlorophyll content, relative water content (RWC), total fresh yield, total dry yield, essential oil (EO) content and essential oil (EO) yield were determined. In both years, plants were harvested during the third week of June at the stage of full flowering. Plants were cut at 5 cm above ground level and then dried in a shaded and ventilated environment for approx. 10 days at a temperature of 25–30°C.

The chlorophyll content was measured using a Dualex Scientific (Force A, Orsay, France) portable Chlorophyll meter. Thirty fully developed leaves were used per plot. The instrument automatically averaged these readings.

The RWC of leaves was estimated using fresh leaves, and the fresh weight (FW) was recorded. The leaves were floated in a falcon tube with distilled water for 24 h. The leaves were then removed from the water and placed on absorbent paper to remove the excess water, and the turgid weight (TW) was recorded. Subsequently, the leaves were dried in an oven for 24 h and the dry weight (DW) was recorded (AlYemeni et al., 2018). The RWC was calculated using the following equation:


$$RWC=\;\frac{FW-DW}{TW-DW}\;\times 100$$

### 2.5 Essential oil extraction

EO content was obtained by hydro distillation of air-dried plant material (500 g) for 3 h in accordance with international guidelines (European Pharmacopoeia, 2008). The EO samples were stored at -18°C. Prior to GC/MS the essential oils obtained during the second growing season were diluted 1:100 with hexane and transferred to GC vials and stored at -18°C.

### 2.6 Essential oil profile, gas chromatography-mass Spectrometry

EO compounds were identified using a HP 6890 gas chromatograph coupled with the quadrupole mass spectrometer HP5972 MSD (Hewlett-Packard, Palo Alto, CA, USA) fitted with a DB5-MS capillary column (30 m × 0.25 mm inner diameter, film thickness: 0.25 μm; Agilent, Palo Alto, CA, USA). Helium was used as carrier gas (average velocity: 42 cm s^-1^), the injector temperature was set to 250°C and the split ratio to 100:1. The temperature program started with 60°C for 4 min, rising to 100°C with 5°C min^-1^ increase, and from 100 to 280°C with 9°C min^-1^. The retention indices of the essential oil compounds were determined in comparison to n-alkane hydrocarbons (retention index standard for GC, Sigma-Aldrich, Vienna, Austria) under the same conditions. The compounds were identified comparing their mass spectra and retention indices to published data. The composition was obtained by peak-area normalization, and the response factor for each compound was considered to equal 1.

### 2.7 Total phenolics, antioxidant activity and rosmarinic acid

#### 2.7.1 Extraction

0.15 g of the finely powdered dry biomass obtained during the second year were extracted with 25 mL aqueous methanol (70%) for 30 minutes in an ultrasonic bath. The extracts were filtered and kept at -18°C until further analysis.

#### 2.7.2 Total phenolics

The total phenolics content was assayed with the Folin-Ciocalteu reagent, following the methodology described by Lamien-Meda et al. (2010). In the wells of the microplate, 5 µL extracts were added to 105 µL distilled water followed by 5 µL of Folin-Ciocalteu reagent, 10 µL Na_2_CO_3_ (35% in distilled water) and again 125 µL distilled water. Caffeic acid (Sigma-Aldrich, Austria; 10 mg in 100 mL milli-Q water) was used as standard. Increasing volumes (0 to 25 µL) of caffeic acid made up to 110 µL with distilled water instead of the samples were used to obtain a calibration curve. A blank was used to correct the readings. Calibration points and samples were pipetted and measured as quadruplicates. After 1 h resting in the dark, the absorbance of the reaction mixture was measured at 750 nm using a microplate reader (i-mark, Bio-Rad, Austria). The results were expressed as milligram caffeic acid equivalents per gram dry weight (mg c.a.e. g^-1^ dw).

#### 2.7.3 Antioxidant activity

Antioxidants react with the stable 2,2-diphenyl-1-picrylhydrazyl radical (DPPH) which is then decolorized. In accordance with the methodology reported by Chizzola et al. (2008), in the wells of the microplate, 5 µL extracts were added to 95 µL methanol and 100 µL of solution (2.2-diphenyl-1-picrylhydrazyl, Sigma-Aldrich, Germany; 0.0038 g in 25 mL methanol). Increasing volumes (0 to 8 µL) of Trolox (0.62 mg mL^-1^ in ethanol) made up to 100 µL with methanol instead of the samples were used to obtain a calibration curve. A preparation consisting of 50 µL Trolox, 50 µL distilled water and 100 µL DPPH reagent (where the DPPH was completely decolorized) was taken as blank and subtracted from all measurements. Calibration points and samples were pipetted and measured as quadruplicates. Discoloration was measured at 490 nm using a microplate reader (i-mark, Bio-Rad, Austria). The results were expressed in milligram trolox equivalents per gram dry weight (mg t.e. g^-1^ dw).

#### 2.7.4 Rosmarinic acid

The content of rosmarinic acid was measured according to Chizzola et al. (2008) using a Waters HPLC system consisting of a 626 pump, a 600S controller, a 717plus autosampler, a column oven operated at 25°C, and a 996-diode array detector (Waters S.A.S, Saint-Quentin, France). The separation was carried out on a Symmetry C18, 5.0 μm particle size, 4.6 × 150 mm column. The mobile phase used was 1% acetic acid/acetontrile 85:15 (solvent A) and methanol (solvent B). The analysis started with a solvent ratio of A/B of 9:1, and a linear gradient was performed to reach 100% B within 30 min. The flow rate was 1.0 mL min^-1^ and the injection volume, 20 μL.

The quantification of rosmarinic acid was done using the external standard method by preparing seven calibration standards ranging from 3.9 to 500 μg ml^-1^ and recording the calibration curve at 330 nm.

### 2.8 Statistical analysis

Statistical analyses were performed using the package MINITAB 19 for Windows. Data were compared using analysis of variance (ANOVA). The difference between means was carried out using Tukey’s test (*p* ≤ 0.05). Before the statistical analysis, all data were tested for normality with a Shapiro–Wilk test, and for homogeneity of variance with Levene’s test.

## 3 Results

### 3.1 Morphological and yield parameters

ANOVA revealed that Frequency factor significantly affected (*p* ≤ 0.01) the RWC during both years (**Table 2**). The highest RWC (81.6% and 83.7%) were always observed in plants treated every two weeks (**Table 2**).

**Table 2:** Influence of Frequency (F), Biostimulant (B) and their interaction on *S. officinalis* plant height, chlorophyll content and relative water content (RWC) over the 2-years study.

Source of variation	Degree of freedom	Plant height [cm]		Chlorophyll content [µg cm^-2^]		RWC [%]	
		I Year	II Year	I Year	II Year	I Year	II Year
Frequency (F)							
1 week		37.9 ± 1.2 ^a^	51.0 ± 1.8 ^a^	35.1 ± 0.7 ^a^	36.4 ± 0.6 ^a^	74.7 ± 0.8 ^b^	76.7 ± 0.8 ^b^
2 weeks		37.8 ± 1.6 ^a^	51.1 ± 1.9 ^a^	35.6 ± 0.7 ^a^	36.6 ± 0.4 ^a^	81.6 ± 0.8 ^a^	83.7 ± 1.1 ^a^
Biostimulant (B)							
C		28.0 ± 1.0 ^c^	38.6 ± 0.8 ^c^	32.5 ± 0.3 ^c^	35.0 ± 0.4 ^c^	76.7 ± 0.4 ^b^	77.7 ± 0.8 ^c^
PH		40.9 ± 0.6 ^a^	56.0 ± 1.5 ^a^	35.1 ± 1.0 ^b^	36.3 ± 0.4 ^bc^	77.8 ± 1.3 ^b^	81.0 ± 1.4 ^ab^
EM		41.8 ± 0.5 ^a^	55.7 ± 0.6 ^a^	33.5 ± 0.5 ^bc^	35.4 ± 0.7 ^c^	77.9 ± 2.5 ^b^	80.3 ± 2.5 ^b^
AN		38.5 ± 0.7 ^b^	52.6 ± 1.1 ^b^	37.8 ± 0.6 ^a^	38.3 ± 1.0 ^a^	77.6 ± 3.3 ^b^	79.9 ± 3.5 ^b^
FA		40.0 ± 0.9 ^ab^	52.3 ± 1.5 ^b^	37.8 ± 0.7 ^a^	37.4 ± 0.6 ^ab^	80.7 ± 1.1 ^a^	82.2 ± 1.4 ^a^
*p*-value							
Frequency	1	0.828	0.893	0.290	0.781	0.000	0.000
Biostimulant	4	0.000	0.000	0.000	0.000	0.000	0.000
F × B	4	0.000	0.000	0.004	0.000	0.000	0.000

Means and standard errors are reported. Values with different letters are significantly different at *p* ≤ 0.05 according to Tukey’s test. C, control; PH, protein hydrolysate; EM, *Ecklonia maxima*; AN, *Ascophyllum nodosum*; FA, fulvic acids.

**Figure 3:**
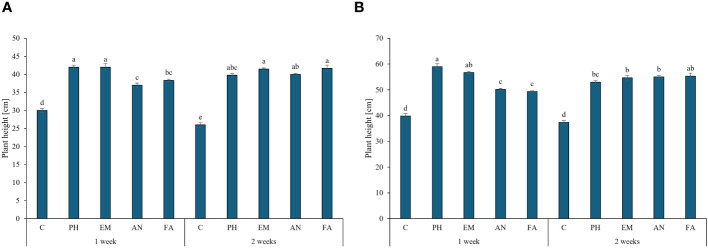
Influence of the interaction Frequency (F) × Biostimulant (B) on *S. officinalis* plant height, in the first year **(A)** and in the second year **(B)**. Means and standard errors are reported. Values with different letters are significantly different at *p* ≤ 0.05 according to Tukey’s test. C, control; PH, protein hydrolysate; EM, *Ecklonia maxima*; AN, *Ascophyllum nodosum*; FA, fulvic acids.

Biostimulant factor and the interaction F × B had significant effect (*p* ≤ 0.01) on plant height, chlorophyll content and RWC (**Table 2**). During the first year, the highest plant heights were recorded in PH- and EM-treated plants every week (42 cm) and in EM- and FA-treated plants every two weeks (41.5 and 41.7 cm, respectively) (**Figure 3A**). During the second year, the highest plant height (59 cm) was recorded in PH-treated plants every week, followed by the values observed by the interactions Frequency 1 × EM and Frequency 2 × FA (**Figure 3B**). During both years, the lowest plant heights were recorded in C-plants (**Figures 3A**, **B**).

**Figure 4:**
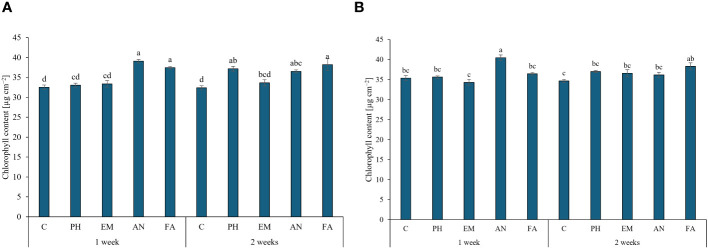
Influence of the interaction Frequency (F) × Biostimulant (B) on *S. officinalis* chlorophyll content, in the first year **(A)** and in the second year **(B)**. Means and standard errors are reported. Values with different letters are significantly different at *p* ≤ 0.05 according to Tukey’s test. C, control; PH, protein hydrolysate; EM, *Ecklonia maxima*; AN, *Ascophyllum nodosum*; FA, fulvic acids.

The application of AN and FA every week and the application of FA every two weeks produced the highest chlorophyll content (values between 37.4 and 39.1 µg cm^-2^), during the first year. The lowest values were observed in C-plants following both frequencies (**Figure 4A**). The interaction Frequency 1 × AN produced the highest chlorophyll content during the second year. The lowest values were recorded by the interactions Frequency 1 × EM and Frequency 2 × C (**Figure 4B**).

**Figure 5:**
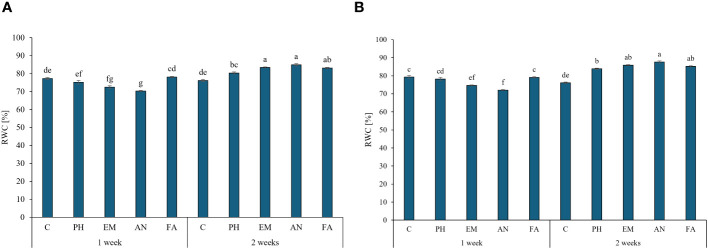
Influence of the interaction Frequency (F) × Biostimulant (B) on *S. officinalis* relative water content (RWC), in the first year **(A)** and in the second year **(B)**. Means and standard errors are reported. Values with different letters are significantly different at *p* ≤ 0.05 according to Tukey’s test. C, control; PH, protein hydrolysate; EM, *Ecklonia maxima*; AN, *Ascophyllum nodosum*; FA, fulvic acids.

During both years, the highest RWC values were observed in EM-, AN- and FA-treated plants every two weeks (values between 83.2% and 87.6%). The lowest values were observed in AN-treated plants every week (**Figures 5A**, **B**).

Statistically analysis showed that frequency factor, biostimulant factor and the interaction F × B had significant effects (*p* ≤ 0.01) on total fresh yield and total dry yield (**Table 3**).

**Table 3:** Influence of Frequency (F), Biostimulant (B) and their interaction on *S. officinalis* total fresh yield and total dry yield over the 2-years study.

Source of variation	Degree of freedom	Total fresh yield [t ha^-1^]		Total dry yield [t ha^-1^]	
		I Year	II Year	I Year	II Year
Frequency (F)						
1 week		3.4 ± 0.2 ^a^	6.5 ± 0.4 ^a^	1.1 ± 0.1 ^a^	2.1 ± 0.1 ^a^
2 weeks		2.6 ± 0.1 ^b^	4.8 ± 0.1 ^b^	0.8 ± 0.0 ^b^	1.4 ± 0.0 ^b^
Biostimulant (B)					
C		2.4 ± 0.1 ^d^	4.4 ± 0.1 ^d^	0.7 ± 0.0 ^d^	1.3 ± 0.1 ^d^
PH		3.4 ± 0.2 ^a^	6.5 ± 0.5 ^a^	1.1 ± 0.1 ^a^	2.0 ± 0.2 ^a^
EM		2.7 ± 0.1 ^c^	5.1 ± 0.1 ^c^	0.9 ± 0.1 ^c^	1.7 ± 0.1 ^c^
AN		3.0 ± 0.2 ^b^	5.7 ± 0.4 ^b^	0.9 ± 0.1 ^c^	1.7 ± 0.1 ^c^
FA		3.4 ± 0.4 ^a^	6.6 ± 0.9 ^a^	1.0 ± 0.1 ^b^	1.9 ± 0.3 ^b^
*p*-value					
Frequency	1	0.000	0.000	0.000	0.000
Biostimulant	4	0.000	0.000	0.000	0.000
F × B	4	0.000	0.000	0.000	0.000

Means and standard errors are reported. Values with different letters are significantly different at *p* ≤ 0.05 according to Tukey’s test. C, control; PH, protein hydrolysate; EM, *Ecklonia maxima*; AN, *Ascophyllum nodosum*; FA, fulvic acids.

**Figure 6:**
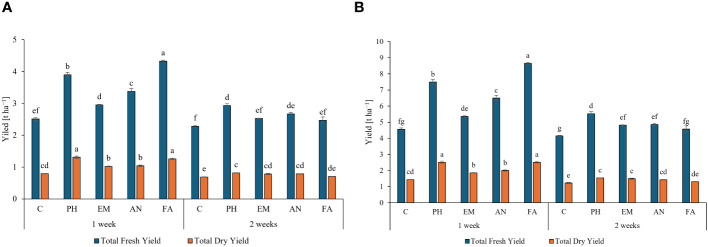
Influence of the interaction Frequency (F) × Biostimulant (B) on *S. officinalis* total fresh yield and total dry yield, in the first year **(A)** and in the second year **(B)**. Means and standard errors are reported. Values with different letters are significantly different at *p* ≤ 0.05 according to Tukey’s test. C, control; PH, protein hydrolysate; EM, *Ecklonia maxima*; AN, *Ascophyllum nodosum*; FA, fulvic acids.

Considering both years, the weekly application of FA produced the highest total fresh yield (4.3 and 8.7 t ha^-1^) and the weekly application of PH and FA generated the highest total dry yields, 1.3 t ha^-1^ during the first year and 2.5 t ha^-1^ during the second year (**Figures 6A, B**). The lowest yield values (fresh and dry) were always observed in C-plants following the frequency 2 weeks (**Figures 6A, B**).

Analysis of variance for EO content and EO yield displayed a significant effect (*p* ≤ 0.01) of frequency, biostimulant and the interaction F × B (**Table 4**).

**Table 4:** Influence of Frequency (F), Biostimulant (B) and their interaction on *S. officinalis* essential oil (EO) content and essential oil (EO) yield over the 2-years study.

Source of variation	Degree of freedom	EO content [%]		EO yield [kg ha^-1^]	
		I Year	II Year	I Year	II Year
Frequency (F)					
1 week		1.23 ± 0.05 ^b^	1.11 ± 0.04 ^b^	13.4 ± 0.9 ^a^	22.9 ± 1.6 ^a^
2 weeks		1.35 ± 0.03 ^a^	1.24 ± 0.03 ^a^	10.3 ± 0.3 ^b^	17.5 ± 0.7 ^b^
Biostimulant (B)					
C		1.30 ± 0.05 ^b^	1.15 ± 0.03 ^bc^	9.7 ± 0.6 ^c^	15.4 ± 1.0 ^d^
PH		1.46 ± 0.02 ^a^	1.32 ± 0.02 ^a^	15.7 ± 1.9 ^a^	27.0 ± 3.1 ^a^
EM		1.28 ± 0.08 ^b^	1.18 ± 0.08 ^b^	11.3 ± 0.2 ^b^	19.5 ± 0.4 ^bc^
AN		1.20 ± 0.05 ^c^	1.10 ± 0.05 ^c^	10.9 ± 0.3 ^b^	18.6 ± 0.7 ^c^
FA		1.22 ± 0.07 ^c^	1.12 ± 0.06 ^c^	11.6 ± 0.8 ^b^	20.5 ± 1.8 ^b^
*p*-value					
Frequency	1	0.000	0.000	0.000	0.000
Biostimulant	4	0.000	0.000	0.000	0.000
F × B	4	0.000	0.000	0.000	0.000

Means and standard errors are reported. Values with different letters are significantly different at *p* ≤ 0.05 according to Tukey’s test. C, control; PH, protein hydrolysate; EM, *Ecklonia maxima*; AN, *Ascophyllum nodosum*; FA, fulvic acids.

**Figure 7:**
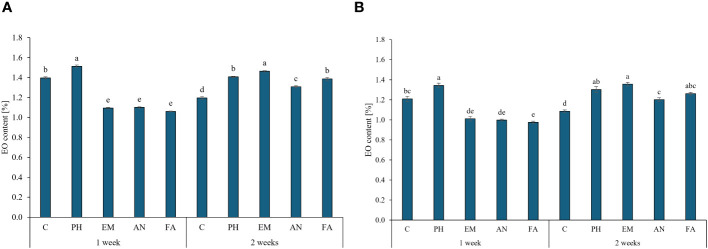
Influence of the interaction Frequency (F) × Biostimulant (B) on *S. officinalis* essential oil (EO) content, in the first year **(A)** and in the second year **(B)**. Means and standard errors are reported. Values with different letters are significantly different at *p* ≤ 0.05 according to Tukey’s test. C, control; PH, protein hydrolysate; EM, *Ecklonia maxima*; AN, *Ascophyllum nodosum*; FA, fulvic acids.

Considering both years, PH-treated plants every week and EM-treated plants every two weeks produced the highest EO content (values between 1.34% and 1.51%). The lowest EO content were obtain in the other biostimulated plants every week (**Figures 7A**, **B**).

**Figure 8:**
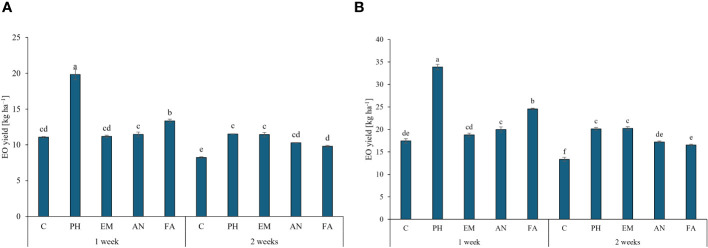
Influence of the interaction Frequency (F) × Biostimulant (B) on *S. officinalis* essential oil (EO) yield, in the first year **(A)** and in the second year **(B)**. Means and standard errors are reported. Values with different letters are significantly different at *p* ≤ 0.05 according to Tukey’s test. C, control; PH, protein hydrolysate; EM, *Ecklonia maxima*; AN, *Ascophyllum nodosum*; FA, fulvic acids.

Regarding the EO yield, the application of PH every week generated the highest values in both years (19.8 kg ha^-1^ during the first years and 33.9 kg ha^-1^ during the second year). The lowest EO yields was obtained in C-plants following frequency 2 (**Figures 8A**, **B**).

### 3.2 Essential oil profile

The results of the analysis of variance of the EO profile are reported in **Table 5**. 44 compounds were identified, and the main components being α-pinene, camphene, β-pinene, 1,8-cineole, α-thujone, β-thujone, camphor, β‐caryophyllene, aromadendrene, α‐humulene, and viridiflorol.

The frequency factor significantly (*p* ≤ 0.05) influenced the percentage of 38 compounds (**Table 5**). Considering only the main components, the highest percentages of α-pinene, camphene, β-pinene, 1,8-cineole, β-Thujone, β‐caryophyllene, aromadendrene were observed in plants treated following frequency 1. In plants treated following frequency 2 the highest percentages of α-thujone, camphor α‐humulene, viridiflorol were measured (**Table 6**).

**Table 5:** Chemical constituents of *S. officinalis* essential oil and *p*-value in response to Biostimulants (B), Frequency (F) and their interaction (B × F).

Peak	RI calc	RI lit	Compounds	Biostimulant (B)	Frequency (F)	Interaction B × F
1	918	919	tricyclene	0.002	0.918	0.290
2	922	923	α-thujene	0.081	0.039	0.618
3	929	933	α-pinene	0.006	0.896	0.458
4	942	952	camphene	0.003	0.989	0.289
5	968	973	sabinene	0.008	0.122	0.198
6	971	981	β-pinene	0.003	0.877	0.198
7	989	991	β-myrcene	0.011	0.912	0.173
8	1002	1005	α -phellandrene	0.051	0.108	0.306
9	1013	1018	α-terpinene	0.010	0.158	0.519
10	1021	1026	*p*-cymene	0.005	0.237	0.597
11	1027	1030	1,8-cineole	0.002	0.721	0.104
12	1036	1040	*cis*-β-ocimene	0.654	0.141	0.318
13	1054	1059	γ-terpinene	0.010	0.201	0.442
14	1062	1069	*trans*-sabinene hydrate	0.427	0.447	0.783
15	1084	1084	α-terpinolene	0.058	0.118	0.339
16	1103	1102	α-thujone	0.005	0.369	0.415
17	1113	1110	β-thujone	0.001	0.911	0.583
18	1133	1139	*trans*-sabinol	0.001	0.435	0.232
19	1140	1143	camphor	0.020	0.428	0.273
20	1160	1165	borneol	0.009	0.472	0.559
21	1172	1178	terpinen-4-ol	0.005	0.277	0.326
22	1185	1185	α-terpineol	0.071	0.204	0.992
23	1286	1285	bornyl acetate	0.087	0.463	0.938
24	1373	1376	α-copaene	0.008	0.419	0.259
25	1375	1375	β-elemene	0.010	0.468	0.089
26	1407	1409	α-gurjunene	0.007	0.375	0.319
27	1428	1428	β‐caryophyllene	0.011	0.092	0.414
28	1434	1436	geranyl acetone	0.004	0.229	0.056
29	1439	1439	aromadendrene	0.010	0.293	0.284
30	1444	1444	α‐caryophyllene	0.014	0.338	0.331
31	1456	1452	α‐humulene	0.005	0.931	0.310
32	1462	1461	allo-aromadendrene	0.487	0.432	0.529
33	1478	1482	citronellyl isobutyrate	0.502	0.162	0.137
34	1490	1490	valencene	0.117	0.371	0.694
35	1492	1493	viridiflorene	0.012	0.305	0.362
36	1496	1502	cuparene	0.014	0.200	0.316
37	1516	1512	γ-cadinene	0.038	0.059	0.115
38	1526	1524	δ-cadinene	0.157	0.028	0.396
39	1573	1575	spathulenol	0.023	0.124	0.460
40	1583	1581	caryophyllene oxide	0.172	0.040	0.804
41	1598	1590	viridiflorol	0.004	0.997	0.192
42	1609	1607	humulene epoxide II	0.043	0.057	0.514
43	1616	1611	epicedrol	0.004	0.997	0.391
44	2070	2056	manool	0.231	0.018	0.677

RI calc, Retention Indices calculated based on C9-C27 n-alkenes from a HP-5MS-column; RI lit, Retention Indices according to literature.

**Table 6:** Influence of Frequency (F) of application on chemical constituents of *S. officinalis* essential oil.

Peak	Compounds	Frequency 1 week [%]	Frequency 2 weeks [%]	*p*-value
1	tricyclene	0.15 ^a^	0.07 ^b^	0.918
2	α-thujene	0.24	0.29	0.039
3	α-pinene	3.44 ^a^	2.25 ^b^	0.896
4	camphene	3.88 ^a^	2.74 ^b^	0.989
5	sabinene	0.06 ^b^	0.12 ^a^	0.122
6	β-pinene	2.71 ^a^	2.16 ^b^	0.877
7	β-myrcene	2.20 ^a^	1.85 ^b^	0.912
8	α -phellandrene	0.10	0.11	0.108
9	α-terpinene	0.40 ^b^	0.54 ^a^	0.158
10	*p*-cymene	0.50 ^b^	0.72 ^a^	0.237
11	1,8-cineole	18.81 ^a^	16.45 ^b^	0.721
12	*cis*-β-ocimene	0.12	0.11	0.141
13	γ-terpinene	0.51 ^b^	0.70 ^a^	0.201
14	*trans*-sabinene hydrate	0.14	0.14	0.447
15	α-terpinolene	0.37	0.42	0.118
16	α-thujone	10.58 ^b^	16.01 ^a^	0.369
17	β-thujone	4.74 ^a^	4.38 ^b^	0.911
18	*trans*-sabinol	0.10 ^a^	0.08 ^b^	0.435
19	camphor	16.06 ^b^	18.51 ^a^	0.428
20	borneol	1.43 ^a^	1.07 ^b^	0.472
21	terpinen-4-ol	0.24 ^b^	0.30 ^a^	0.277
22	α-terpineol	0.33	0.29	0.204
23	bornyl acetate	0.71	0.56	0.463
24	α-copaene	0.30 ^a^	0.17 ^b^	0.419
25	β-elemene	0.11 ^a^	0.08 ^b^	0.468
26	α-gurjunene	1.94 ^a^	0.87 ^b^	0.375
27	β‐caryophyllene	8.82 ^a^	7.20 ^b^	0.092
28	geranyl acetone	0.49 ^a^	0.28 ^b^	0.229
29	aromadendrene	3.55 ^a^	2.00 ^b^	0.293
30	α‐caryophyllene	0.38 ^a^	0.22 ^b^	0.338
31	α‐humulene	5.74 ^b^	8.16 ^a^	0.931
32	allo-aromadendrene	0.47	0.64	0.432
33	citronellyl isobutyrate	0.17	0.16	0.162
34	valencene	0.26	0.2	0.371
35	viridiflorene	0.08 ^a^	0.03 ^b^	0.305
36	cuparene	1.42 ^a^	0.91 ^b^	0.200
37	γ-cadinene	0.09 ^a^	0.08 ^b^	0.059
38	δ-cadinene	0.31	0.28	0.028
39	spathulenol	1.15 ^a^	0.53 ^b^	0.124
40	caryophyllene oxide	0.77	0.62	0.04
41	viridiflorol	3.11 ^b^	4.67 ^a^	0.997
42	humulene epoxide II	0.75 ^a^	0.48 ^b^	0.057
43	epicedrol	0.25 ^b^	0.40 ^a^	0.997
44	manool	0.5	0.59	0.018

Means are reported. Values with different letters are significantly different at *p* ≤ 0.05 according to Tukey’s test.

The Biostimulant factor significantly (*p* ≤ 0.05) influenced the percentage of α-thujene, δ-cadinene, caryophyllene oxide and manool (**Table 7**). The highest α-thujene (0.34%) content was observed in PH-treated plants, this value was statistically similar to those obtained in EM-, AN- and FA treated plants. The lowest content (0.20%) was found in C-plants (**Table 7**). In C-plants, the highest content of δ-cadinene (0.36%) and caryophyllene oxide (1.02%) was observed, while the lowest content was found in PH-treated plants (δ-cadinene 0.25% and caryophyllene oxide 0.48%). The values obtained with the other foliar biostimulants were statistically similar to each other (**Table 7**). The highest manool content (0.80%) was observed in C-plants, followed by those statistically similar obtained in AN-, PH- and EM-treated plants. In FA-treated plants, the lowest manool content was observed (0.38%) (**Table 7**).

**Table 7:** Influence of Biostimulant (F) on chemical constituents of *S. officinalis* essential oil.

Peak	Compounds	C [%]	PH [%]	EM [%]	AN [%]	FA [%]	*p*-value
1	tricyclene	0.12	0.09	0.10	0.11	0.12	0.918
2	α-thujene	0.20 ^b^	0.34 ^a^	0.28 ^ab^	0.25 ^ab^	0.25 ^ab^	0.039
3	α-pinene	2.84	2.72	2.57	2.93	3.16	0.896
4	camphene	3.33	3.33	3.17	3.27	3.45	0.989
5	sabinene	0.05	0.14	0.11	0.09	0.07	0.122
6	β-pinene	2.35	2.59	2.35	2.43	2.47	0.877
7	β-myrcene	1.94	2.12	1.99	2.01	2.07	0.912
8	α -phellandrene	0.09	0.12	0.11	0.10	0.10	0.108
9	α-terpinene	0.37	0.57	0.51	0.45	0.45	0.158
10	*p*-cymene	0.47	0.72	0.69	0.59	0.58	0.237
11	1,8-cineole	16.87	18.14	17.39	17.61	18.14	0.721
12	*cis*-β-ocimene	0.10	0.13	0.12	0.11	0.12	0.141
13	γ-terpinene	0.51	0.73	0.68	0.60	0.60	0.201
14	*trans*-sabinene hydrate	0.12	0.15	0.15	0.15	0.14	0.447
15	α-terpinolene	0.33	0.45	0.43	0.38	0.38	0.118
16	α-thujone	10.38	15.69	14.83	12.75	12.81	0.369
17	β-thujone	4.59	4.49	4.56	4.55	4.62	0.911
18	*trans*-sabinol	0.10	0.08	0.09	0.10	0.08	0.435
19	camphor	15.69	18.09	18.47	16.92	17.27	0.428
20	borneol	1.36	1.04	1.18	1.31	1.34	0.472
21	terpinen-4-ol	0.23	0.28	0.29	0.28	0.28	0.277
22	α-terpineol	0.32	0.27	0.30	0.33	0.33	0.204
23	bornyl acetate	0.76	0.53	0.58	0.68	0.63	0.463
24	α-copaene	0.31	0.18	0.20	0.25	0.24	0.419
25	β-elemene	0.11	0.09	0.10	0.11	0.10	0.468
26	α-gurjunene	2.06	0.94	1.14	1.50	1.41	0.375
27	β‐caryophyllene	9.53	6.94	7.51	8.35	7.73	0.092
28	geranyl acetone	0.51	0.28	0.33	0.39	0.42	0.229
29	aromadendrene	3.84	1.97	2.37	2.95	2.74	0.293
30	α‐caryophyllene	0.41	0.21	0.26	0.32	0.30	0.338
31	α‐humulene	6.40	7.36	7.26	6.98	6.74	0.931
32	allo-aromadendrene	0.53	0.37	0.41	0.45	1.04	0.432
33	citronellyl isobutyrate	0.19	0.15	0.16	0.17	0.16	0.162
34	valencene	0.29	0.17	0.20	0.24	0.25	0.371
35	viridiflorene	0.09	0.03	0.05	0.06	0.06	0.305
36	cuparene	1.58	0.85	1.03	1.26	1.13	0.200
37	γ-cadinene	0.11	0.07	0.08	0.09	0.08	0.059
38	δ-cadinene	0.36 ^a^	0.25 ^b^	0.28 ^ab^	0.31 ^ab^	0.28 ^ab^	0.028
39	spathulenol	1.49	0.42	0.66	0.89	0.74	0.124
40	caryophyllene oxide	1.02 ^a^	0.48 ^b^	0.58 ^ab^	0.70 ^ab^	0.68 ^ab^	0.040
41	viridiflorol	3.93	3.99	3.78	3.97	3.78	0.997
42	humulene epoxide II	1.00	0.38	0.52	0.61	0.55	0.057
43	epicedrol	0.32	0.34	0.32	0.32	0.32	0.997
44	manool	0.80 ^a^	0.51 ^ab^	0.51 ^ab^	0.53 ^ab^	0.38 ^b^	0.018

Means are reported. Values with different letters are significantly different at *p* ≤ 0.05 according to Tukey’s test. C, control; PH, protein hydrolysate; EM, *Ecklonia maxima*; AN, *Ascophyllum nodosum*; FA, fulvic acids.

The analysis of variance showed that the interaction factor did not statistically affect the percentage of EO compounds (**Table 6**).

### 3.3 Total phenolics, antioxidant activity and rosmarinic acid

Analysis of variance revealed that biostimulant (B) and frequency (F) factors had significant effects (*p* ≤ 0.05) on total phenolic, antioxidant activity and rosmarinic acid (**Table 8**).

**Table 8:** Effect of Biostimulant (B), Frequency (F) and their interaction on total phenolic, antioxidant activity and rosmarinic acid.

	Total Phenolic [mg c.a.e. g^-1^ dw]	Antioxidant Activity [mg t.e. g^-1^ dw]	Rosmarinic Acid [%]
Frequency (F)			
1 week	57.0 ± 2.8 ^b^	70.0 ± 6.5 ^b^	1.1 ± 0.7 ^b^
2 weeks	62.3 ± 3.5 ^a^	94.2 ± 5.3 ^a^	1.5 ± 0.6 ^a^
Biostimulant (B)			
C	76.0 ± 2.1 ^a^	108.2 ± 2.3 ^a^	2.4 ± 0.1 ^a^
PH	68.3 ± 1.9 ^b^	65.3 ± 12.4 ^b^	0.8 ± 0.4 ^b^
EM	58.4 ± 1.9 ^c^	78.3 ± 8.0 ^ab^	1.1 ± 0.0 ^b^
AN	43.9 ± 1.4 ^e^	81.6 ± 9.2 ^ab^	1.3 ± 0.6 ^b^
FA	51.8 ± 1.2 ^d^	77.4 ± 11.4 ^ab^	1.0 ± 0.4 ^b^
*p*-value			
F	0.000	0.003	0.016
B	0.000	0.017	0.000
F × B	0.001	0.612	0.666

Means and standard errors are reported. Values with different letters are significantly different at *p* ≤ 0.05. c.a.e., Caffeic acid equivalent; t.e., Trolox equivalent; C, control; PH, protein hydrolysate; EM, *Ecklonia maxima*; AN, *Ascophyllum nodosum*; FA, fulvic acids.

The highest total phenolic (76.0 mg c.a.e. g^-1^) and rosmarinic acid (2.4%) values were observed in Control plants. The lowest total phenolic value was observed in FA-treated plants, recorded as 24.2 mg c.a.e. g^-1^ lower than the highest content (**Table 8**). The lowest rosmarinic acid values were observed in all biostimulated plants (between 0.8% and 1.3%) (**Table 8**).

Considering antioxidant activity, the highest value (108.2 mg t.e. g^-1^) was obtained in C-plants, statistically similar to those obtained in EM-, AN- and FA-treated plants (**Table 8**).

Regardless of the biostimulant factor, sage plants showed higher total phenolic, antioxidant activity and rosmarinic acid levels following Frequency 2.

**Figure 9:**
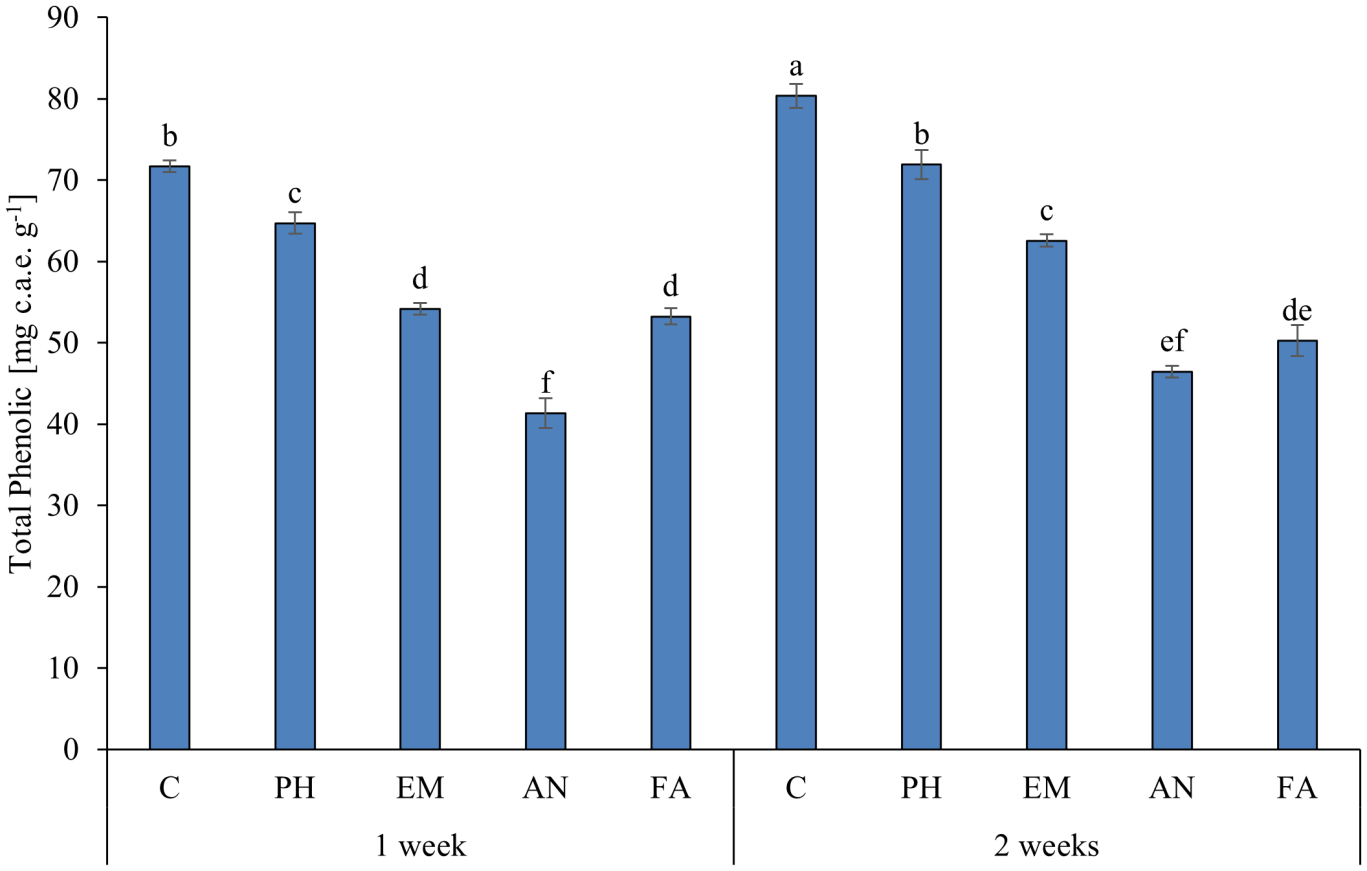
Influence of the interaction F × B on total phenolic. Means and standard errors are reported. Values with different letters are significantly different at *p* ≤ 0.05 according to Tukey’s test. c.a.e., Caffeic acid equivalent; C, control; PH, protein hydrolysate; EM, *Eklonia maxima*; AN, *Ascophyllum nodosum*; FA, fulvic acids.

Statistical analysis highlighted that the interaction B × F has a significant effect (*p* ≤ 0.01) only on total phenolic content (**Table 8**). As reported in **Figure 3**, the highest total phenolic content (80.4 mg c.a.e. g^-1^) was observed in C-plants following Frequency 2. The lowest total phenolic value (41.3 mg c.a.e. g^-1^) was recorded in AN-treated plants following Frequency 1. This value was statistically similar to those obtained in AN-treated plants following Frequency 2 (**Figure 9**).

## 4 Discussion

Nowadays, the cultivation of medicinal and aromatic plants has garnered increasing attention, necessitating the improvement of agronomic practices to maximize yields and harvest quality in a sustainable and logical manner (Sharifi-Rad et al., 2017; Rao et al., 2022). In addition to being used in the kitchen to add flavors to food, the food, cosmetic, and chemical sectors are increasingly using the MAPs to look for particular properties and/or compounds that can be found on various MAPs (Bernardini et al., 2018; Giannenas et al., 2020; Ovidi et al., 2021). Because of this, it’s essential to develop and assess the impact that various agronomic techniques have on the chemical composition and qualitative characteristics of the extracts obtained from MAPs, in addition to refining the agronomic technique to boost yields (Elansary et al., 2019; Kosakowska et al., 2019; Giannoulis et al., 2020; Shahrajabian et al., 2021). Increased crop yields with the use of uncommon agricultural practices are one of the modern agriculture’s goals (Di Miceli et al., 2022; Licata et al., 2022; Krein et al., 2023).

This study demonstrated how the foliar application of various biostimulants, a technique that is being used more and more and has produced excellent results on a variety of species, has improved the agronomic performance of *S. officinalis* biologically cultivated in a Mediterranean environment. In addition, both qualitative and chemical measures were impacted by the foliar treatment of biostimulants.

The quantity and frequency of applications, which may affect nutrient uptake and plant metabolic processes, are additional factors to consider (Bulgari et al., 2019; Li et al., 2022). At the meantime, the most efficient and financially beneficial course of action must be determined while keeping in mind that a higher number of applications translate into higher costs for farmers (Kocira et al., 2020; Li et al., 2022).

Over the course of the two years of the test, the largest heights were achieved with the foliar application of protein hydrolysates and *Ecklonia maxima*. Protein hydrolysates are combinations of amino acids and peptides that, when applied to plants, can cause a range of physiological reactions that support growth, improve the yield and quality of the product, and strengthen the plants’ resistance to heat stress, salinity, drought, and nutritional stress (Schaafsma, 2009; Colla et al., 2014). The positive effect of applying *E. maxima* may be related to the content of macro- and micronutrients, sterols, polysaccharides, betaines, and, additionally, enhancing-promoting compounds (auxins, cytokinins, gibberellins) (Tarakhovskaya et al., 2007; Briceño-Domínguez et al., 2014; Hernandez-Herrera et al., 2014; Arioli et al., 2015).

The height values are comparable to those found in the research conducted by Es-sbihi et al. (2021), which measured heights between 40 and 50 cm after applying salicylic acid foliar treatments to sage plants. Similar results were found by Soltanbeigi et al. (2021) on *S. officinalis* cultivated in greenhouses and fertilized with various methods.

During both years, treatment with *Ascophyllum nodosum* and fulvic acids increased the amount of chlorophyll in sage leaves. Numerous studies (Abdel-Rahman and Abdel-Kader, 2020; Ciriello et al., 2022; Rasouli et al., 2022; Farruggia et al., 2024) have reported that plants treated with foliar biostimulants exhibited greater levels of chlorophyll in their leaves when compared to untreated plants. The values observed in this study agree with the chlorophyll content measured by Aslani et al. (2023) in *S. officinalis* under drought stress and inoculation treatments. The chlorophyll content of sage plants exposed to increasing nitrogen dosages showed a similar tendency into Khammar et al. work (Khammar et al., 2021).

Reduced cell turgor and relative water content (RWC) in water-stressed plants lead to decreased cell elongation and development, which in turn reduces leaf area. When antioxidant defenses and reactive oxygen species (ROS) are out of equilibrium in water-stressed plants, oxidative stress results (Mohammadi-Cheraghabadi et al., 2021).

A lower number of foliar seaweed applications produced an increase in the relative water content of the leaf. The application of the same biostimulants following the frequency 1 causes a significant decrease in RWC. Auxins, cytokinins, and gibberellins - enhancing-promoting compounds in algae extracts – in excessive quantity can be phytotoxic to plants and cause negative effects on the physiological processes and on the assimilation of water and nutrients (Illera-Vives et al., 2022). Due to the content of macro and micro-nutrients and plant hormones, the correct application of seaweed extracts can improve root growth and development and, at the same time, can maintain a balanced water content and a correct transpiration rate (Azizi et al., 2024).

During the second year of the research, the highest yield values were obtained thanks to the greater development of plants at the third year of cultivation. In general, a more frequent biostimulant application has allowed to obtain higher biomass and EO yield. During both years, the highest biomass yields (fresh and dry) were observed in plants treated with protein hydrolysate and fulvic acid. Peptide and amino acid content of protein hydrolysates is the primary cause of their biostimulatory activities (Di Miceli et al., 2023). Amino acids are used by plants for many different purposes, such as the synthesis of substances with high biological activity, the generation of energy, and the biosynthesis of proteins (Paul et al., 2019; Rouphael et al., 2022). Protein hydrolysates applied on leaves or on roots can alter the phyllosphere or rhizosphere’s microbial community (Colla et al., 2017). Microorganisms in the rhizosphere and phyllosphere may release enzymes that break down peptides into smaller pieces that function as signaling molecules to promote plant development (Malécange et al., 2023). Fulvic acids are known to facilitate the absorption and translocation of micro- and macronutrients. For this reason, their application has been connected to increases in crop productivity and quality (Colombo et al., 2012; Aytaç et al., 2022). Additionally, these compounds positively influence several molecular functions, such as the production of proteins, photosynthetic activity, and enzyme activity (Nardi et al., 2021; Farruggia et al., 2024). Their application can boost the concentration of nitrogen, phosphorus, and potassium, as well as the content of photosynthetic pigments, carotenoids, and total phenols (Zanin et al., 2019; Aytaç et al., 2022). The yield obtained in this study are in line with those observed by Ostadi et al. (2022) in *S. officinalis* under drought stress and treated with TiO_2_ nanoparticles and arbuscular mycorrhizal fungi. Also, Giannoulis et al. (2021) measured similar fresh yield in *S. officinalis* cultivated in Greece with different plant density and fertilization methods.

It is well known that the synthesis of secondary metabolites is affected by endogenous and exogenous factors (Rajabi et al., 2014; Ben Akacha et al., 2023). Several research report that the application of biostimulants can improve the beneficial qualities of plants by acting as elicitors for secondary metabolites, such as essential oils (Rahmani Samani et al., 2019; The et al., 2021; Ciriello et al., 2024). During the two-year study, the EO content ranged between 1.0% and 1.5%. These values are in line with those obtained by Ostadi et al. (2022) (0.6%-1.5%), Tarraf et al. (2017) (1.0%-1.2%), Govahi et al. (2015) (0.8%-2.1%), Bagdat et al. (2017) (1.2%-1.6%), Es-sbihi et al. (2021) (1.2%-2.8%) measured under different conditions. Otherwise, Geneva et al. (2010) observed a lower EO content (0.4%-0.5%) in *S. officinalis* fertilized with foliar NPK + microelements. Rahmani Samani et al. (2019) reported EO content from 0.6% to 0.8% after L-phenylalanine foliar application and inoculation of the roots of seedlings with plant grown promoters. Rioba et al. (2015) also obtained EO content lower than 1.0% in *S. officinalis* under different levels of nitrogen, phosphorus, and irrigation.

As reported by several authors (Delamare et al., 2007; Abu-Darwish et al., 2013; Russo et al., 2013; Rajabi et al., 2014; La Bella et al., 2015; Mot et al., 2022), due to factors including geographic location, time of year, climate, genetic variations, phenological stages, sampling techniques, and extraction techniques, the percentage of EO constituents varies greatly. In this work, 44 constituents were identified, with the following being the most representative (above 2%): 1,8-cineole (average 17.6%), camphor (average 17.3%), α-thujone (average 13.3%), β‐caryophyllene (average 8.0%), α‐humulene (average 7.0%), β-thujone (average 4.6%), viridiflorol (average 3.9%), camphene (average 3.3%), aromadendrene (average 2.8%), α-pinene (average 2.8%), β-pinene (average 2.4%).

In accordance with other studies (Russo et al., 2013; Rajabi et al., 2014; Jažo et al., 2023; Schmiderer et al., 2023) the most represented class are monoterpenes, and 1.8-cineole, camphor and α-thujone. The presence of these compounds in sage essential oils has been linked to its antibacterial, antifungal, anti-inflammatory, antiseptic, antiscabies, and antisyphilitic qualities (Lahlou et al., 2002; Delamare et al., 2007; Abu-Darwish et al., 2013; Kim et al., 2014; Jažo et al., 2023).

Results of interaction effects of frequency × biostimulant reported in **Supplementary Table S1** (not presented because no statistically significant difference observed) indicated that the highest concentrations of 1,8-cineole (21.0%) was observed in plants treated with fulvic acids following the frequency 1 week (**Supplementary Table S1**), an increase of 4.2% point compared to control plants. The other biostimulants foliar application following the same frequency allowed to obtain and increase between 1.8–2.1% point compared to control plants. The camphor percentage increased by 5.1% point thanks to the application of fulvic acids following the frequency 2 compared to untreated plants. In general, the frequency 2 and biostimulant application allowed to obtain camphor percentage higher than 3.0–5.1% point compared to those observed in control plants (**Supplementary Table S1**). Similar trend has been observed in α-thujone content. In particular, the application of the four different biostimulant following the frequency 2 permitted to register an increase of camphor percentage between 1.8% and 3.9% point. In addition to the increase of biomass production, the use of biostimulants may promote the production of monoterpenes in sage leaves. The biostimulant may affect the activity of enzymes and the regulation of genes in the metabolic pathway linked to the creation of secondary metabolites (Vosoughi et al., 2018). Regarding other monoterpenes, such as α-pinene, camphene, β-pinene, the application of the four different biostimulant following the frequency 1 allowed to obtain a small increase in percentage content compared to control plant. β-Thujone values were similar in all treatment and ranged between 4.3–4.9%. β‐Caryophyllene percentage was 9.5% in control plants following both frequency and in FA-treated plants following frequency 1, while in other treated plants ranged from 6.0% to 8.7%, with a particular decrease following the frequency 2. A similar trend was observed for the sesquiterpene aromadendrene. Regarding the sesquiterpene α‐humulene, relevant increase of the content was measured in biostimulated plants following the frequency 2, with an increase of 1.5–2.9% point compared to control plants. Viridiflorol content decreased in biostimulated plant following frequency 1 but the percentage increased in biostimulated plant following frequency 2 compared to control plants.

Biostimulant application can change certain metabolic pathways and biochemical characteristics in plants (Amer et al., 2021). These substances have the potential to alter the pathway of secondary metabolites, impact plastid and chlorophyll, modify stress tolerance, and eventually manipulate the amount and quality of EO (Alkharpotly et al., 2024). According to Salehi et al. (2019), biostimulant helps plants to better access nutrients which promote the growth and division of glandular trichomes, and EO channels. In addition, these products can increase the photosynthetic activity of enzymes and precursors of EO (Rehman et al., 2016). In this study, biostimulant application resulted in an increase in monoterpenes and in a decrease in the synthesis of sesquiterpenes and diterpenes. In MAPs, phytohormones and phytohormone-like substances are involved in the stimulation and synthesis of volatile chemicals as well as other compounds (Pirbalouti et al., 2019). The increase in total content of monoterpenes could be linked to the presence within the biostimulants of growth promoting substance such as auxine, cytochines and giberellic acid, involved in the metabolic pathways of monoterpenes (Tarraf et al., 2017).

Contrary to what is observed with EO content, regarding total phenolic content, antioxidant activity and rosmarinic acid content, the highest values were always observed in control plants. In biostimulated plants a decrease of these parameters was observed compared to control plants. It is commonly known that stressful conditions affect MAPs capacity to create secondary metabolites (Figueiredo et al., 2008; Tawfeeq et al., 2016; Kulak et al., 2020; Chaski et al., 2023; Farruggia et al., 2023). Presumably, the foliar biostimulant treatments and the rains that fell during the last stage of the cycle, before the sample collection, helped the sage plants evade stress situations and consequently lower the production of some secondary metabolites, like phenols and rosmarinic acid. However, a variety of writers (Elansary et al., 2019; Bonini et al., 2020; Saia et al., 2021; Rahimi et al., 2022) have noted that when various species are exposed to microbial and non-microbial biostimulants the quantity of secondary metabolites increases. Our data suggested to avoid generalization of effects on secondary metabolites and take stress or stress-relief into consideration as well.

## 5 Conclusion

The results of the present study highlight some positive effects of biostimulants foliar application on morphological, productive and yield parameters of sage plants under rainfed conditions. All biostimulants produced an improvement in plant growth and yields compared to control plants. In particular, the more frequent application of fulvic acid and protein hydrolysate allowed to obtain the highest biomass and EO yields. The highest EO content was observed in plant treated every week with protein hydrolysate. In the EO obtained from biostimulated plants every week was registered the highest 1,8-cineole percentage. The highest percentage increases in the content of α-thujone and camphor were observed in biostimulated plants following frequency 2. Otherwise, the biostimulant application has caused a decrease in total phenolic, antioxidant activity and rosmarinic acid values, compared to untreated plants. Sage yields can be increased by the foliar application of biostimulants, and this practice is advised particularly for organic cultivation. Further research is needed to better understand the mechanisms of action of biostimulants on medicinal and aromatic plants primary and secondary metabolism.

## Data availability statement

The raw data supporting the conclusions of this article will be made available by the authors, without undue reservation.

## Author contributions

DF: Conceptualization, Data curation, Formal analysis, Investigation, Methodology, Software, Validation, Writing – original draft, Writing – review & editing. GD: Formal analysis, Validation, Visualization, Writing – review & editing, Supervision. ML: Conceptualization, Methodology, Supervision, Validation, Visualization, Writing – original draft, Writing – review & editing. CL: Funding acquisition, Project administration, Resources, Writing – review & editing, Supervision. FS: Investigation, Software, Writing – original draft. JN: Conceptualization, Formal analysis, Methodology, Supervision, Validation, Visualization, Writing – original draft, Writing – review & editing.
