# Supplementary material for: Foliar application of various biostimulants produces contrasting response on yield, essential oil and chemical properties of organically grown sage (Salvia officinalis L.)
Source: Front Plant Sci. 2024 Jun 28;15:1397489. doi: 10.3389/fpls.2024.1397489 (PMC11248988; doi:10.3389/fpls.2024.1397489)
Supplement: Supplementary file 1 [file Table_1.docx]

Supplementary Material

# Supplementary Tables

**Table S1.** Influence of the interaction F × B on chemical constituents of *S. officinalis* essential oil.

|  |  | **Frequency 1 week** | | | | |  | **Frequency 2 weeks** | | | | |
| --- | --- | --- | --- | --- | --- | --- | --- | --- | --- | --- | --- | --- |
| **Peak** | **Compounds** | **C**  **[%]** | **PH**  **[%]** | **EM**  **[%]** | **AN**  **[%]** | **FA**  **[%]** |  | **C**  **[%]** | **PH**  **[%]** | **EM**  **[%]** | **AN**  **[%]** | **FA**  **[%]** |
| 1 | tricyclene | 0.12 | 0.12 | 0.14 | 0.14 | 0.20 |  | 0.11 | 0.06 | 0.05 | 0.08 | 0.03 |
| 2 | α-thujene | 0.20 | 0.29 | 0.24 | 0.25 | 0.21 |  | 0.20 | 0.39 | 0.32 | 0.24 | 0.29 |
| 3 | α-pinene | 2.85 | 3.15 | 3.30 | 3.57 | 4.31 |  | 2.84 | 2.29 | 1.83 | 2.29 | 2.01 |
| 4 | camphene | 3.42 | 3.63 | 3.91 | 3.74 | 4.68 |  | 3.23 | 3.03 | 2.43 | 2.80 | 2.22 |
| 5 | sabinene | 0.04 | 0.12 | 0.07 | 0.07 | 0.00 |  | 0.07 | 0.16 | 0.15 | 0.10 | 0.14 |
| 6 | β-pinene | 2.40 | 2.68 | 2.68 | 2.70 | 3.11 |  | 2.29 | 2.49 | 2.01 | 2.17 | 1.83 |
| 7 | β-myrcene | 1.92 | 2.15 | 2.21 | 2.21 | 2.53 |  | 1.96 | 2.09 | 1.78 | 1.81 | 1.61 |
| 8 | α -phellandrene | 0.09 | 0.10 | 0.10 | 0.10 | 0.09 |  | 0.08 | 0.14 | 0.12 | 0.10 | 0.12 |
| 9 | α-terpinene | 0.35 | 0.47 | 0.41 | 0.43 | 0.33 |  | 0.38 | 0.66 | 0.61 | 0.46 | 0.58 |
| 10 | *p*-cymene | 0.42 | 0.58 | 0.53 | 0.55 | 0.39 |  | 0.52 | 0.86 | 0.84 | 0.62 | 0.77 |
| 11 | 1,8-cineole | 16.8 | 18.6 | 18.9 | 18.9 | 21.0 |  | 16.97 | 17.68 | 15.92 | 16.37 | 15.32 |
| 12 | *cis*-β-ocimene | 0.10 | 0.12 | 0.12 | 0.12 | 0.13 |  | 0.10 | 0.14 | 0.12 | 0.11 | 0.10 |
| 13 | γ-terpinene | 0.50 | 0.63 | 0.55 | 0.58 | 0.45 |  | 0.52 | 0.83 | 0.80 | 0.62 | 0.76 |
| 14 | *trans*-sabinene hydrate | 0.13 | 0.15 | 0.13 | 0.14 | 0.13 |  | 0.11 | 0.16 | 0.16 | 0.15 | 0.14 |
| 15 | α-terpinolene | 0.35 | 0.40 | 0.39 | 0.38 | 0.32 |  | 0.32 | 0.50 | 0.48 | 0.38 | 0.45 |
| 16 | α-thujone | 9.93 | 13.50 | 11.07 | 11.11 | 7.28 |  | 10.84 | 17.87 | 18.59 | 14.39 | 18.34 |
| 17 | β-thujone | 4.69 | 4.62 | 4.85 | 4.68 | 4.84 |  | 4.49 | 4.35 | 4.26 | 4.43 | 4.39 |
| 18 | *trans*-sabinol | 0.11 | 0.09 | 0.10 | 0.10 | 0.11 |  | 0.08 | 0.07 | 0.08 | 0.09 | 0.05 |
| 19 | camphor | 16.3 | 17.0 | 16.9 | 15.7 | 14.3 |  | 15.1 | 19.2 | 20.0 | 18.1 | 20.2 |
| 20 | borneol | 1.42 | 1.20 | 1.43 | 1.41 | 1.70 |  | 1.31 | 0.89 | 0.92 | 1.22 | 0.98 |
| 21 | terpinen-4-ol | 0.21 | 0.26 | 0.25 | 0.27 | 0.22 |  | 0.24 | 0.29 | 0.33 | 0.29 | 0.35 |
| 22 | α-terpineol | 0.34 | 0.28 | 0.32 | 0.35 | 0.34 |  | 0.30 | 0.25 | 0.27 | 0.32 | 0.31 |
| 23 | bornyl acetate | 0.81 | 0.58 | 0.68 | 0.72 | 0.76 |  | 0.72 | 0.48 | 0.48 | 0.63 | 0.49 |
| 24 | α-copaene | 0.30 | 0.24 | 0.29 | 0.29 | 0.39 |  | 0.32 | 0.11 | 0.11 | 0.21 | 0.08 |
| 25 | β-elemene | 0.10 | 0.10 | 0.11 | 0.11 | 0.13 |  | 0.12 | 0.07 | 0.08 | 0.10 | 0.06 |
| 26 | α-gurjunene | 2.03 | 1.43 | 1.84 | 1.82 | 2.59 |  | 2.08 | 0.45 | 0.44 | 1.18 | 0.22 |
| 27 | β‐caryophyllene | 9.54 | 7.87 | 8.51 | 8.71 | 9.47 |  | 9.52 | 6.00 | 6.51 | 7.98 | 6.00 |
| 28 | geranyl acetone | 0.50 | 0.36 | 0.46 | 0.43 | 0.71 |  | 0.53 | 0.20 | 0.20 | 0.36 | 0.13 |
| 29 | aromadendrene | 3.68 | 2.74 | 3.41 | 3.40 | 4.50 |  | 4.01 | 1.19 | 1.34 | 2.49 | 0.97 |
| 30 | α‐caryophyllene | 0.39 | 0.30 | 0.38 | 0.36 | 0.48 |  | 0.42 | 0.13 | 0.15 | 0.28 | 0.13 |
| 31 | α‐humulene | 6.34 | 6.70 | 5.70 | 5.82 | 4.12 |  | 6.47 | 8.01 | 8.81 | 8.15 | 9.36 |
| 32 | allo-aromadendrene | 0.52 | 0.41 | 0.46 | 0.45 | 0.51 |  | 0.54 | 0.32 | 0.36 | 0.44 | 1.56 |
| 33 | citronellyl isobutyrate | 0.17 | 0.17 | 0.17 | 0.16 | 0.18 |  | 0.21 | 0.14 | 0.15 | 0.18 | 0.14 |
| 34 | valencene | 0.28 | 0.20 | 0.26 | 0.25 | 0.31 |  | 0.30 | 0.14 | 0.14 | 0.23 | 0.18 |
| 35 | viridiflorene | 0.09 | 0.06 | 0.08 | 0.07 | 0.11 |  | 0.10 | 0.00 | 0.03 | 0.04 | 0.01 |
| 36 | cuparene | 1.52 | 1.11 | 1.37 | 1.41 | 1.71 |  | 1.64 | 0.59 | 0.68 | 1.11 | 0.54 |
| 37 | γ-cadinene | 0.10 | 0.08 | 0.09 | 0.09 | 0.10 |  | 0.11 | 0.06 | 0.07 | 0.09 | 0.05 |
| 38 | δ-cadinene | 0.35 | 0.29 | 0.29 | 0.31 | 0.32 |  | 0.37 | 0.22 | 0.26 | 0.31 | 0.24 |
| 39 | spathulenol | 1.48 | 0.70 | 1.06 | 1.05 | 1.44 |  | 1.50 | 0.14 | 0.26 | 0.73 | 0.03 |
| 40 | caryophyllene oxide | 0.99 | 0.57 | 0.71 | 0.73 | 0.83 |  | 1.05 | 0.38 | 0.45 | 0.67 | 0.53 |
| 41 | viridiflorol | 3.99 | 3.47 | 2.87 | 3.30 | 1.92 |  | 3.86 | 4.51 | 4.69 | 4.63 | 5.63 |
| 42 | humulene epoxide II | 0.98 | 0.52 | 0.69 | 0.68 | 0.88 |  | 1.03 | 0.25 | 0.35 | 0.54 | 0.23 |
| 43 | epicedrol | 0.30 | 0.30 | 0.22 | 0.26 | 0.16 |  | 0.33 | 0.39 | 0.42 | 0.39 | 0.47 |
| 44 | manool | 0.86 | 0.42 | 0.42 | 0.47 | 0.33 |  | 0.74 | 0.59 | 0.59 | 0.60 | 0.43 |

Means are reported. C = control; PH = protein hydrolysate; EM = *Eklonia maxima*; AN = *Ascophyllum nodosum*; FA = fulvic acids.

**
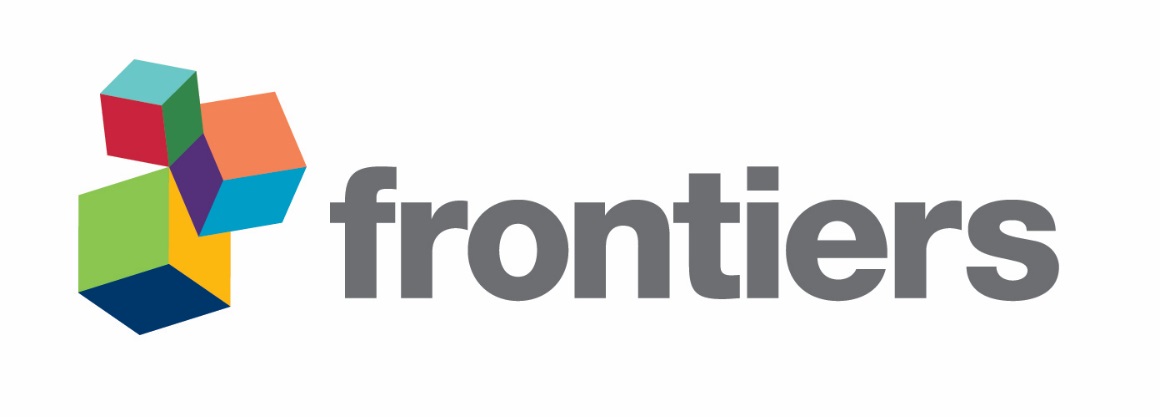
**
